# System‐dependent image distortion related to gantry positions of a 0.35 T MRgRT: Characterization and the corresponding correction

**DOI:** 10.1002/acm2.13826

**Published:** 2022-11-10

**Authors:** Shanti Marasini, Benjamin Quinn, Mike Cole, Rocco Flores, Taeho Kim

**Affiliations:** ^1^ Department of Radiation Oncology Washington University School of Medicine St. Louis Missouri USA; ^2^ Department of the Modus Medical Modus Medical Devices Inc. London Ontario Canada

**Keywords:** distortion correction, geometric image distortion, MR‐guided radiotherapy

## Abstract

**Purpose:**

MR‐guided radiotherapy with high accuracy treatment planning requires addressing MR imaging artifacts that originate from system imperfections. This work presents the characterization and corresponding correction of gantry‐related imaging distortions including geometric distortion and isocenter shift in a 0.35 T magnetic resonance imaging (MRI)‐guided radiotherapy (MRgRT) system using distortion vector fields (DVFs).

**Methods:**

Two phantoms, the magnetic resonance imaging distortion in 3D (MRID^3D^) phantom and the Fluke phantom, along with a human volunteer were imaged at different gantry angles on a 0.35 T MR‐Linac. The geometric distortion and isocenter shift were characterized for both phantom images. DVFs with a field of view extended beyond the physical boundary of the MRID^3D^ phantom were extracted from images taken at 30° gantry angle increments, with vendor‐provided distortion correction turned on and off (DstOff). These extended DVFs were then applied to the relevant phantom images to correct their geometric distortions and isocenter shift at the respective gantry angles. The extended DVFs produced from the MRID^3D^ phantom were also applied to Fluke phantom and human MR images at their respective gantry angles. The resampled images were evaluated using structural similarity index measure (SSIM) comparison with the vendor corrected images from the MRgRT system.

**Results:**

Geometric distortion with “mean (± SD) distortion” of 3.2 ± 0.02, 2.9 ± 0.02, and 1.8 ± 0.01 mm and isocenter shift (±SD) of 0.49 ± 0.3, 0.05 ± 0.2, and 0.01 ± 0.03 mm were present in the DstOff MRID^3D^ phantom images in right–left (RL), anterior–posterior (AP), and superior–inferior (SI) directions, respectively. After resampling the originally acquired images by applying extended DVFs, the distortion was corrected to 0.18 ± 0.02, 0.09 ± 0.01, 0.15 ± 0.01 mm, and isocenter shift was corrected to 0.14 ± 0.05, −0.02 ± 0.04, and −0.07 ± 0.05 mm in RL, AP, and SI directions, respectively. The Fluke phantom average geometric distortion with “mean (± SD) distortion” of 2.7 ± 0.1 mm was corrected to 0.2 ± 0. 1 mm and the average isocenter shift (± SD) of 0.51 ± 0.2 mm, and 0.05 ± 0.03 was corrected to −0.08 ± 0.03 mm, and −0.05 ± 0.01 in RL and AP directions, respectively. SSIM (mean ± SD) of the original images to resampled images was increased from 0.49 ± 0.02 to 0.78 ± 0.01, 0.45 ± 0.02 to 0.75 ± 0.01, and 0.86 ± 0.25 to 0.98 ± 0.08 for MRID^3D^ phantom, Fluke phantom, and human MR images, respectively, for all the gantry angles compared to the vendor corrected images.

**Conclusion:**

The gantry‐related MR imaging distortion including geometric distortion and isocenter shift was characterized and a corresponding correction was demonstrated using extended DVFs on 0.35 T MRgRT system. The characterized gantry‐related isocenter shift can be combined with geometric distortion correction to provide a technique for the correction of the full system‐dependent distortion in an MRgRT system.

## 1 INTRODUCTION

Magnetic resonance imaging (MRI)‐guided radiotherapy (MRgRT), an integrated treatment system of linear accelerator (Linac) with magnetic resonance guidance (MRI), is a promising approach in radiotherapy which allows real‐time tumor imaging and tracking simultaneously during treatment delivery.^1^, ^2^, ^3^, ^4^ Compared with computed tomography (CT) approaches, MRI is superior for tumor localization in radiotherapy because of its excellent soft‐tissue contrast, resolution, and structure identification without any ionizing radiation.^5^, ^6^, ^7^ However, as with any MRI, one of the challenges in MRgRT is dealing with its systematic distortion. These image distortions could lead to suboptimal treatment planning with inaccurate dose calculation and beam gating.^8^, ^9^, ^10^ For high fidelity MRgRT, spatial accuracy is critical for accurate treatment execution, thus characterization of those MR image distortions is important.

MR image distortion usually originates from hardware imperfections such as main magnetic field (*B*_o_) inhomogeneity, gradient nonlinearity, imperfect shimming (active/passive), and eddy current.^10^, ^11^, ^12^ These system defects typically cause geometric distortion in MR images, spatial shift of imaging volumes, and signal heterogeneity. When utilizing MRI for radiotherapy systems additional sources of distortions are introduced. This is due to the imperfect radiofrequency shielding for the Linac component. The MR‐Linac gantry contains a large amount of ferromagnetic material that can induce gradient nonlinearity and eddy currents which cause fluctuation in central frequency. The change in central frequency causes imaging isocenter shift.^13^, ^14^ Noticeable image distortions have been reported due to the impact of the gantry position on MRgRT system.^13^, ^14^, ^15^, ^16^, ^17^ The magnetic field homogeneity and spatial integrity have been studied for multiple gantry angles for 0.35 T MR‐^60^Co and 0.35 T MR‐Linac systems. Both of the MRgRT systems have field homogeneity of less than 5 ppm and spatial integrity distortion of less than 2 mm within a 175 mm radius.^18^ Additional studies at multiple gantry angles have shown that the position of the gantry can vary imaging isocenter position by nearly 2 mm.^16^, ^18^, ^19^, ^20^ Recently, most modern MRgRT systems allow for active shimming and both online and offline application of a vendor correction function into the system to decrease the gradient nonlinearity and magnetic field inhomogeneity effects, but the imaging artifacts such as isocenter variation at different gantry position remains unremedied yet.^21^ Therefore, a single home gantry position, where the imaging system is tuned at commissioning, is still recommended for acquiring clinical imaging with the system.

Several comprehensive quality assurance (QA) programs were launched for characterizing the imaging performance of different MRgRT systems using in‐house volumetric phantoms.^13^, ^19^, ^20^, ^21^, ^22^, ^23^ However, most of the phantoms have been heavy and complicated to set up for regular application. Recently, Lewis et al. used the lightweight magnetic resonance imaging distortion in 3D (MRID^3D^) phantom, and phantom‐dependent geometric distortion analysis software produced by Modus Medical Devices Inc. (Modus QA) to study the imaging distortion and isocenter shifts for multiple gantry angles and to generate distortion vector fields (DVFs) for the correction of the system‐dependent image distortion.^24^ However, there were some limitations in the Lewis et al. study: (1) Because the signal‐generating region of the phantom lies outside the DVF boundary, the resulting DVF could not be used to correct the acquired phantom image. Consequently, DVFs produced from the MRID^3D^ phantom were applied to an independent grid phantom image with a small field of view (FOV) for verification. (2) The imaging isocenter shift was not adjusted after characterization due to the resampling software only resampling using the geometric distortion DVF and not the measured phantom‐isocenter offset.

In this study, we characterized gantry‐related MR imaging geometric distortion and isocenter shift on an institutional 0.35 T MR‐Linac system using the MRID^3D^ phantom using both vendor‐corrected (DstOn) and uncorrected (DstOff) images. In the post‐characterization, we streamlined the system‐dependent correction of the MR imaging distortion including geometric distortion and isocenter shift by utilizing DVFs with an extended FOV extracted from the MRID^3D^ phantom images at various gantry angles of a 0.35 T MR‐Linac system. The resampled images were re‐characterized in the MRID^3D^ geometric distortion analysis software for verification. The extended DVFs were also applied to independent Fluke phantom images and human abdominothoracic images to independently verify the correction.

## 2 METHODS

All the images were acquired on a 0.35 T ViewRay MRIdian MR‐Linac system in MRI QA mode. MRI images were acquired and analyzed for 12 different gantry positions from 0° to 330° at a 30° interval. The system baseline was set at Gantry 300°, as this is the gantry position recommended by the vendor for clinical imaging.

### 2.1 Geometric imaging phantoms

Two phantoms were utilized for MR imaging. The first phantom imaged was the QUASAR™ MRID^3D^ cylindrical geometric distortion phantom; herein referred to as the MRID^3D^ phantom (Modus QA, London, Ontario, Canada) with the dimension of 391 mm in length and 394 mm in diameter. The phantom has a closed surface with 25 L air‐filled space, containing an array of 1502 machined fiducials filled with T_1_‐contrast paraffinic mineral oil. All the fiducial points are placed with 18 mm uniform spacing around the phantom and each fiducial is 6 mm long with a diameter of 5 mm, with the exception of six positioning fiducials that are 8 mm in diameter. These fiducials define a cylindrical boundary 294 mm in length and 343.5 mm in diameter. Distortion within the boundary is calculated using harmonic analysis.^25^ The volumetric 3D images were acquired in axial orientation using the clinical TrueFISP sequence in MRI‐QA mode with: TR/TE: 3.4/1.4 ms, flip angle: 60°, FOV: 450 × 450 × 360 mm^3^, imaging matrix: 274 × 274 × 206, voxel size 1.5 × 1.5 × 1.5 mm^3^, read out bandwidth (rBW): 570 Hz/Px.

The second phantom was the 2D Fluke 76–907 Uniformity and Linearity water, spatial integrity phantom (referred to as the Fluke phantom) doped with 15 mM CuSO_4_ (HP Manufacturing, Cleveland, OH) with the outer dimension of 330 × 330 × 100 mm with a small bubble filled with a solution attached on the surface of the phantom. The phantom is used to examine the geometric integrity of the images. All the images were acquired in axial positions using the clinical TrueFISP sequence in MRI‐QA mode with: TR/TE: 3.4/1.4 ms, flip angle: 60°, FOV: 450 × 450 × 90 mm^3^, imaging matrix 270 × 270 × 60, voxel size 1.5 × 1.5 × 1.5 mm^3^, and rBW: 534 Hz/Px.

**FIGURE 1:**
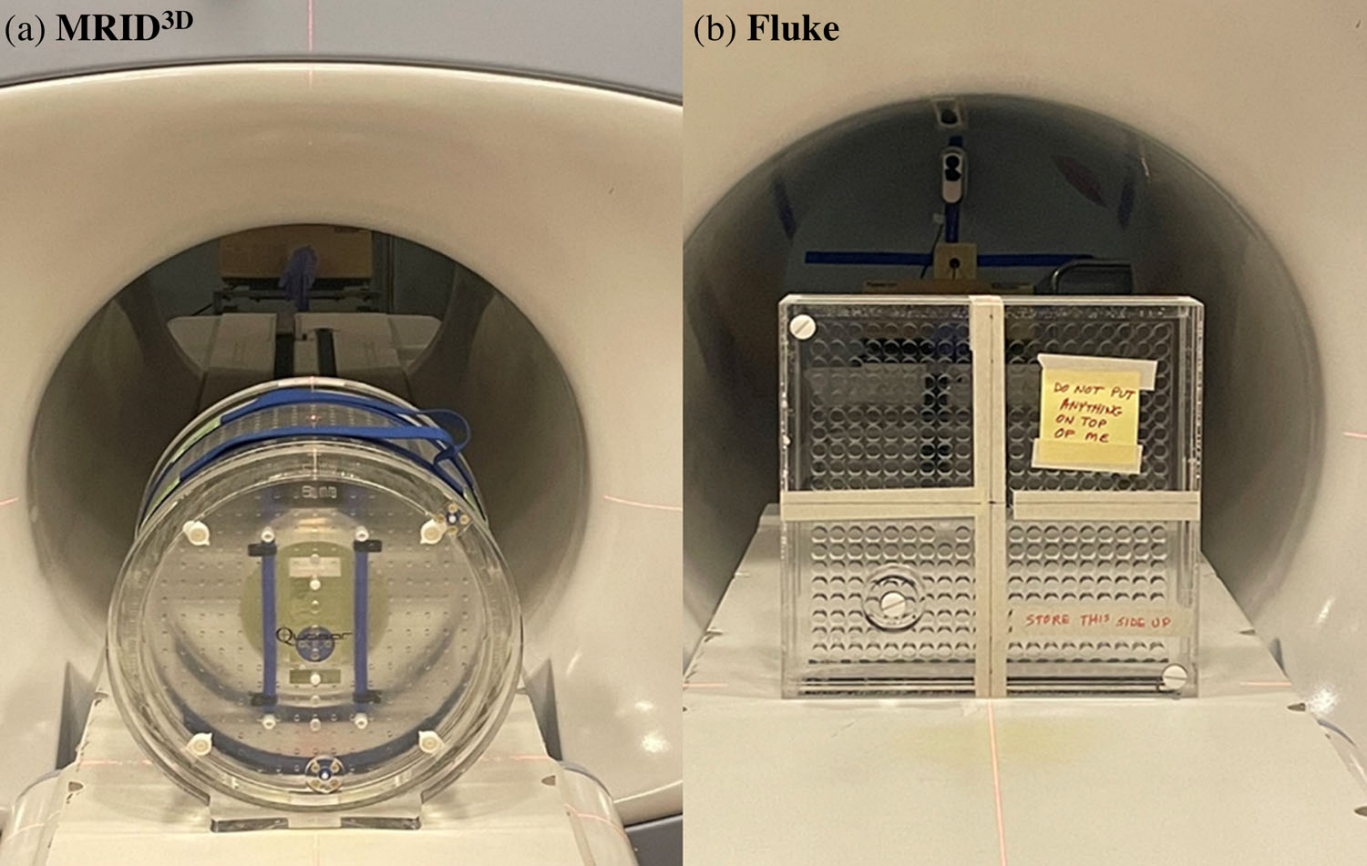
Images of the (a) MRID^3D^ phantom and (b) Fluke phantom on the 0.35 T MR‐Linac.

**FIGURE 2:**
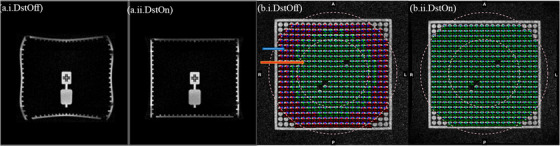
(a) The sagittal view of the MRID^3D^ phantom with the onboard distortion correction turned (i) Off (DstOff) and (ii) On (DstOn) and (b) the axial view of the Fluke phantom with (i) DstOff and (ii) DstOn. In the Fluke phantom, the circular dots indicate the distortion analysis regions for the phantom and the blue and orange arrows indicate the 175 and 100 mm radius analysis regions, respectively.

The images were acquired with the onboard Siemens distortion correction function turned on and off (DstOn and DstOff) and the phase encoding in the anterior–posterior (AP) direction for both the phantoms. The system onboard distortion correction function baseline was calibrated and set at gantry 300° according to the vendor's instruction. Figure 1 shows the phantom images and Figure 2 displays the MR image of the axial planes of both phantoms with DstOff and DstOn.

The volumetric 3D images acquired from the MRID^3D^ phantom were imported to the QUASAR™ MRID^3D^ geometric distortion analysis software. The software produced principal component error values based on harmonic analysis of the boundary fiducials as well as imaging isocenter alignments using analysis of a central orientation structure. The software reports the resultant DVF's mean and maximum distortion values along with the imaging isocenter alignments on the three primary axes. Similarly, the 2D images from Fluke phantom were analyzed using ViewRay's SpatialIntegrityAnalysis 2D software. The software compared the centroid of each circular marker in the grid to the expected location within two analysis spheres of 100 mm and 175 mm radii and reported as a mean and standard deviation of positional error for spatial integrity in a single plane. All geometric distortion and isocenter‐shift values recorded for both the phantoms were relative to the gantry angle of 300°.

### 2.2 Human image acquisition

Human images were acquired using the clinical TrueFISP sequence with TR/TE: 3.8/1.6 ms, flip angle: 60°, FOV 450 × 450 × 240 mm, imaging matrix: 276 × 276 × 80, rBW: 385 Hz/Px, and voxel size 1.6 × 1.6 × 3 mm^3^ with total acquisition time: 17.18 s. The images were acquired at gantry 300° with the onboard Siemens distortion correction function turned on and off (DstOn and DstOff) and the phase encoding in the AP direction.

### 2.3 Geometric distortion correction

The FOV of the DVFs were extended beyond the boundary defined by the MRID^3D^ phantom fiducials using pre‐release software from Modus QA. The software calculates spherical harmonic coefficients from the measured fiducials and reconstructs distortion values at locations beyond the boundary defined by the fiducials. The resulting DVF (herein referred to as the extended DVF) defined a cylinder of length 402 mm and diameter 451.5 mm. Harmonic coefficients up to degree 9 were used for the calculation of the additional distortion points. The extended DVFs were produced for both DstOn and DstOff MRID^3D^ phantom images at each gantry angle and used to resample the phantom images using the System Distortion Resampler software (Modus QA, London, Ontario, Canada). Along with the distortion, the imaging isocenter shift was also corrected using the System Distortion Resampler software.

The extent of imaging isocenter shift along the *x*, *y*, and *z* axes was calculated relative to the isocenter position of the image acquired at gantry 300° where the institutional MRgRT system has been calibrated. The detected values for each image were entered into the System Distortion Resampler software along with the DVFs and applied to the respective images. The resampled MR images were then re‐characterized through their respective software for additional evaluation. The resampled images were compared with both the original images and the onboard Siemens distortion corrected images for geometric distortion correction verification. The structural similarity index measure (SSIM) was calculated using the System Distortion Resampler software comparing DstOff original and resampled images at each gantry angle to the respective DstOn vendor corrected images. The SSIM of DstOff original and resampled images at each gantry angle was also compared with the baseline reference image, that is, DstOn image at gantry angle 300° where the shimming was calibrated during system upgrade. The human image correction was also evaluated using the SSIM between the DstOff images and the resampled DstOff images relative to the DstOn images as further validation of the resampling process.

## 3 RESULTS

### 3.1 Gantry‐dependent geometric distortion

The mean distortion for all the measured gantry angles of the MRID^3D^ phantom images acquired with both DstOff and DstOn is shown in Tables 1 and 2. The highest geometric distortion was seen at the gantry angle of 90° for DstOff scans with the “mean (± SD) distortion” values of 3.2 ± 0.02, 3.0 ± 0.02, and 1.8 ± 0.01 mm in the anterior–posterior (AP), superior–inferior (SI), and right–left (RL) directions, respectively. The maximum distortion values at the gantry angle of 90° DstOff scans were 19.9, 19.2, and 9.4 mm in the AP, SI, and RL directions, respectively. The geometric distortion values were mostly corrected with the onboard Siemens distortion correction function turned on. The “mean (± SD) distortion” value for DstOn scans at the gantry angle of 90° were 0.25 ± 0.03, 0.30 ± 0.01, and 0.27 ± 0.01 mm in the AP, SI, and RL directions, respectively. The maximum distortion value at a gantry angle of 90° was 7.5, 2.2, and 2.2mm in the AP, SI, and RL directions, respectively. The “mean (± SD) distortion” values at a gantry angle 300° for DstOff scans were 3.1 ± 0.02, 2.9 ± 0.02, and 1.8 ± 0.01 mm in the AP, SI, and RL directions, respectively. The “mean (± SD) distortion”’ values at a gantry angle of 300° for DstOn scans were 0.32 ± 0.03, 0.33 ± 0.01, and 0.29 ± 0.01 mm in the AP, SI, and RL directions, respectively. The maximum distortion value at a gantry angle of 300° for DstOff scans was 19.5, 18.9, and 8.9 mm and for DstOn scans was 1.9, 2.3, and 1.7 mm in the AP, SI, and RL directions, respectively.

**TABLE 1:** MRID^3D^ phantom images mean and the maximum (Max) geometric distortion error for DstOff original and resampled images at all gantry angles

	**DstOff (original)**						**DstOff (resampled)**					
	**RL**		**AP**		**SI**		**RL**		**AP**		**SI**	
**Gantry (°)**	**Mean**	**Max**	**Mean**	**Max**	**Mean**	**Max**	**Mean**	**Max**	**Mean**	**Max**	**Mean**	**Max**
0	3.18	20.0	2.89	18.8	1.82	9.11	0.21	1.02	0.09	1.39	0.15	2.2
30	3.18	20.0	2.93	18.7	1.83	9.31	0.2	0.84	0.09	1.44	0.16	2.1
60	3.17	19.4	2.91	18.7	1.81	8.84	0.18	7.06	0.09	1.63	0.14	1.45
90	3.21	19.9	2.99	19.2	1.85	9.39	0.16	0.92	0.12	1.37	0.2	1.39
120	3.18	19.6	2.91	18.7	1.8	8.74	0.17	1.82	0.1	2.02	0.17	1.6
150	3.18	20.0	2.91	19.0	1.84	9.61	0.15	0.93	0.1	1.87	0.19	2.92
180	3.18	19.7	2.93	18.1	1.91	8.92	0.19	2.44	0.12	4.89	0.11	1.41
210	3.16	19.3	2.94	19.3	1.84	9.57	0.22	1.9	0.1	1.92	0.14	2.03
240	3.15	20.0	2.91	18.1	1.8	9.09	0.21	1.27	0.09	1.29	0.13	1.29
270	3.14	19.7	2.94	19.4	1.82	9.25	0.17	1.08	0.08	1.7	0.13	2.22
300	3.14	19.5	2.91	18.9	1.82	8.92	0.14	0.93	0.1	1.7	0.16	1.74
330	3.16	19.2	2.9	19.0	1.8	8.77	0.18	0.94	0.08	1.53	0.15	1.41

Abbreviations: RL, right–left; AP, anterior–posterior; SI, superior–inferior.

All the geometric distortion error values are in mm.

**TABLE 2:** MRID^3D^ phantom images mean and the maximum geometric distortion error for DstOn original and resampled images at all gantry angles

	**DstOn (original)**						**DstOn (resampled)**					
	**RL**		**AP**		**SI**		**RL**		**AP**		**SI**	
**Gantry (°)**	**Mean**	**Max**	**Mean**	**Max**	**Mean**	**Max**	**Mean**	**Max**	**Mean**	**Max**	**Mean**	**Max**
0	0.34	2.58	0.34	2.05	0.27	1.39	0.2	2.88	0.05	1.28	0.11	0.74
30	0.31	1.7	0.36	1.88	0.27	1.53	0.23	1.14	0.07	1.34	0.13	1.54
60	0.3	7.58	0.33	1.89	0.28	1.45	0.26	6.83	0.07	1.18	0.11	0.63
90	0.25	2.03	0.3	1.99	0.27	2.27	0.16	1.08	0.05	0.79	0.15	1.64
120	0.28	2.01	0.32	2.12	0.28	1.62	0.19	1.71	0.06	2.18	0.15	1.23
150	0.27	2	0.32	2.11	0.29	1.74	0.16	1.05	0.07	1.06	0.17	1.34
180	0.29	2.62	0.35	2.02	0.24	1.56	0.2	2.99	0.05	0.96	0.08	0.81
210	0.36	2.07	0.34	2.22	0.27	1.65	0.24	2.2	0.06	1.73	0.11	1.1
240	0.38	2.23	0.32	1.98	0.26	1.82	0.22	1.17	0.06	1.12	0.11	0.58
270	0.34	2.52	0.3	2.22	0.26	1.97	0.19	1.01	0.07	1.52	0.11	2.24
300	0.32	1.94	0.33	2.29	0.29	1.78	0.15	0.55	0.06	1.25	0.13	0.6
330	0.32	2.31	0.32	2.01	0.25	1.93	0.18	0.65	0.03	1.67	0.09	1.11

Abbreviations: RL, right–left; AP, anterior–posterior; SI, superior–inferior.

All the geometric distortion error values are in mm.

The isocenter shift values for each gantry angle in RL, AP, and SI directions are shown in Tables 3 and 4. Relative to the gantry angle of 300°, the maximum isocenter shift of 1 and 0.96 mm for DstOff and DstOn scans, respectively, occurred at a gantry angle of 120° for the RL direction.

**TABLE 3:** MRID^3D^ phantom image isocenter shift for DstOff original and resamples images

	**DstOff (original)**			**DstOff (resampled)**		
**Gantry (°)**	**RL**	**AP**	**SI**	**RL**	**AP**	**SI**
0	0.3	−0.24	0	0.16	−0.07	−0.1
30	0.57	−0.28	−0.02	0.11	−0.05	−0.15
60	0.82	−0.2	0.02	0.19	−0.09	−0.05
90	0.9	−0.08	−0.01	0.21	−0.06	−0.12
120	1	0.07	−0.01	0.22	−0.04	−0.13
150	0.9	0.2	−0.01	0.17	0.01	−0.13
180	9.7	0.38	−0.07	0.16	0.04	0.01
210	0.4	0.4	0.03	0.15	−0.01	−0.09
240	0.19	0.34	0.01	0.14	0.01	−0.07
270	0.03	0.2	0.04	0.12	0.04	−0.04
300	0	0	0	0	0	0
330	0.07	−0.17	0.04	0.12	−0.07	−0.01

Abbreviations: RL, right–left; AP, anterior–posterior; SI, superior–inferior.

All the imaging isocenter‐shift values are in mm

**TABLE 4:** MRID^3D^ phantom image isocenter shift for DstOn original and resamples images

	**DstOn (original)**			**DstOn (resampled)**		
**Gantry (°)**	**RL**	**AP**	**SI**	**RL**	**AP**	**SI**
0	0.3	−0.2	0	0.18	0.07	−0.1
30	0.56	−0.28	−0.02	0.15	−0.05	−0.15
60	0.82	−0.2	0.01	0.2	0.07	−0.08
90	0.9	−0.08	−0.01	0.18	−0.05	−0.12
120	1	0.07	−0.01	0.27	−0.01	−0.14
150	0.9	0.23	−0.01	0.22	0.02	−0.13
180	0.7	0.38	0.07	0.22	0.03	0.05
210	0.4	0.4	0.03	0.2	0.01	−0.09
240	0.19	0.34	0.01	0.19	0.05	−0.07
270	0.03	0.3	0.04	0.16	0.05	−0.02
300	0	0	0	0	0	0
330	0.07	−0.17	0.0	0.16	−0.06	−0.02

RL, right–left; AP, anterior–posterior; SI, superior–inferior.

All the imaging isocenter‐shift values are in mm

The geometric distortion of the Fluke phantom images acquired with DstOff and DstOn at various gantry positions are shown in Table 5. The “mean (± SD) distortion” at a gantry angle of 300° for DstOff and DstOn is 2.7 ± 0.1 and 0.30 ± 0.03 mm, respectively. The maximum distortion value at the gantry angle of 300° for DstOff and DstOn images are 5.2 and 0.66 mm, respectively. The highest mean distortion occurred at gantry angle 150° with the DstOff scan with “mean (± SD) distortion” value of 2.7 ± 0.1 mm. The maximum distortion value at gantry 150° for DstOff scan was 5.5 mm. The isocenter shift value for each gantry angle in AP and RL positions are shown in Table 5. The maximum isocenter shift of 0.96 mm for both DstOff and DstOn scans, respectively, occurred at a gantry angle of 120° for the RL direction (Table 6).

**TABLE 5:** Fluke phantom images mean geometric distortion error for DstOff original and resampled images and DstOn original and resampled images at all gantry angles

	**DstOff (original)**		**DstOff (resampled)**		**DstOn (Original)**		**DstOn (resampled)**	
**Gantry (°)**	**Mean**	**Max**	**Mean**	**Max**	**Mean**	**Max**	**Mean**	**Max**
0	2.73	5.44	0.23	0.83	0.34	0.79	0.19	0.68
30	2.77	5.94	0.21	0.54	0.35	0.80	0.18	0.51
60	2.63	5.52	0.21	0.7	0.38	1.06	0.17	0.69
90	2.74	5.54	0.24	1.06	0.31	0.79	0.18	1.0
120	2.72	5.47	0.23	0.71	0.31	0.73	0.18	0.54
150	2.76	5.53	0.24	0.55	0.3	0.77	0.18	0.58
180	2.69	5.74	0.22	0.85	0.34	0.81	0.17	0.68
210	2.75	5.75	0.23	0.81	0.33	0.91	0.18	0.74
240	2.69	5.37	0.22	0.71	0.34	0.83	0.19	0.58
270	2.76	5.62	0.22	0.56	0.28	0.87	0.17	0.46
300	2.72	5.26	0.23	0.57	0.3	0.66	0.18	0.59
330	2.74	5.79	0.21	0.54	0.33	0.91	0.18	0.48

**TABLE 6:** Fluke phantom images isocenter‐shift for DstOff original and resampled images and DstOn original and resampled images at all gantry angles

	**DstOff (original)**		**DstOff (resampled)**		**DstOn (original)**		**DstOn (resampled)**	
**Gantry (°)**	**RL**	**AP**	**RL**	**AP**	**RL**	**AP**	**RL**	**AP**
0	0.33	−0.29	−0.12	0.03	0.33	−0.28	0.2	0.05
30	0.58	−0.3	−0.13	0.01	0.58	−0.31	−0.21	0.02
60	0.8	−0.28	−0.16	0.05	0.8	−0.27	−0.23	0.05
90	0.92	−0.12	−0.07	−0.16	0.92	−0.13	−0.17	0.01
120	0.96	0.07	−0.1	−0.05	0.97	0.1	−0.17	−0.08
150	0.9	0.27	−0.02	−0.14	0.9	0.29	−0.17	−0.08
180	0.73	0.35	−0.11	−0.11	0.75	0.34	−0.2	−0.02
210	0.5	0.4	−0.2	−0.16	0.5	0.4	−0.23	−0.04
240	0.28	0.34	−0.1	−0.04	0.27	0.33	−0.2	−0.03
270	0.07	0.2	−0.08	−0.1	0.08	0.2	−0.17	−0.01
300	0	0	0	0	0	0	0	0
330	0.11	−0.02	0.2	0.03	0.09	−0.15	0.18	−0.02

Abbreviations: RL, right–left; AP, anterior–posterior.

All the imaging isocenter‐shift values are in mm.

### 3.2 Distortion correction

#### 3.2.1 Self‐image correction

**FIGURE 3:**
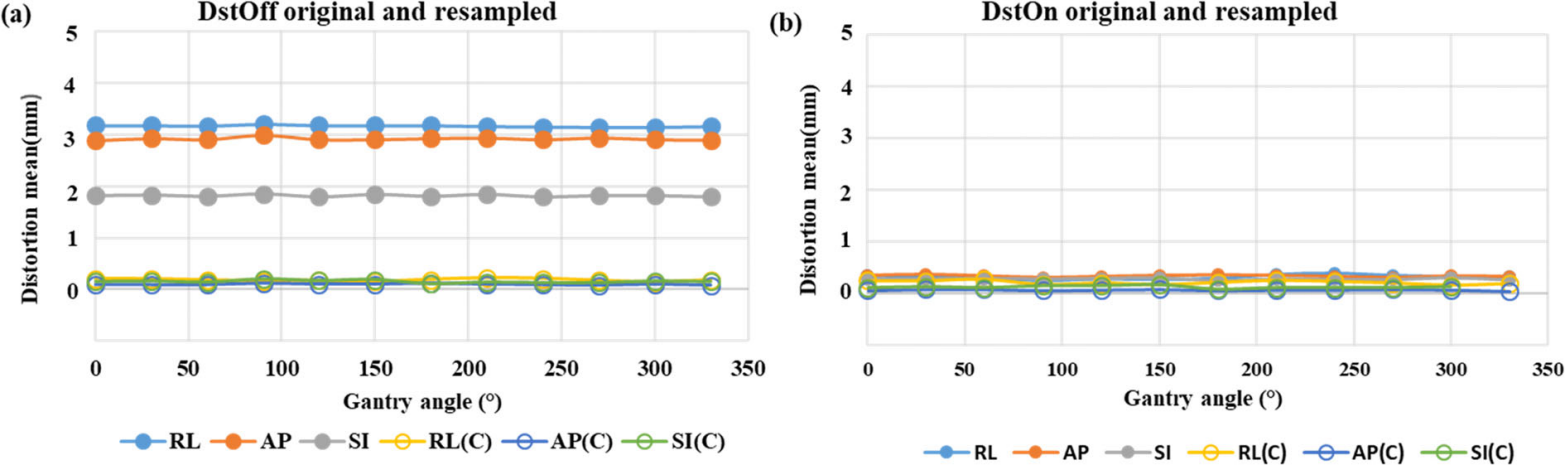
Mean distortion using MRID^3D^ phantom. The mean distortion values of the original and resampled images on the right–left (RL), anterior–posterior (AP), and superior–inferior (SI) directions across all gantry angles. Plots show the values of (a) distortion correction off (DstOff) with distortion correction off resampled (DstOff (R)) and (b) distortion correction on (DstOn) with distortion correction on resampled (DstOn(R)).

**FIGURE 4:**
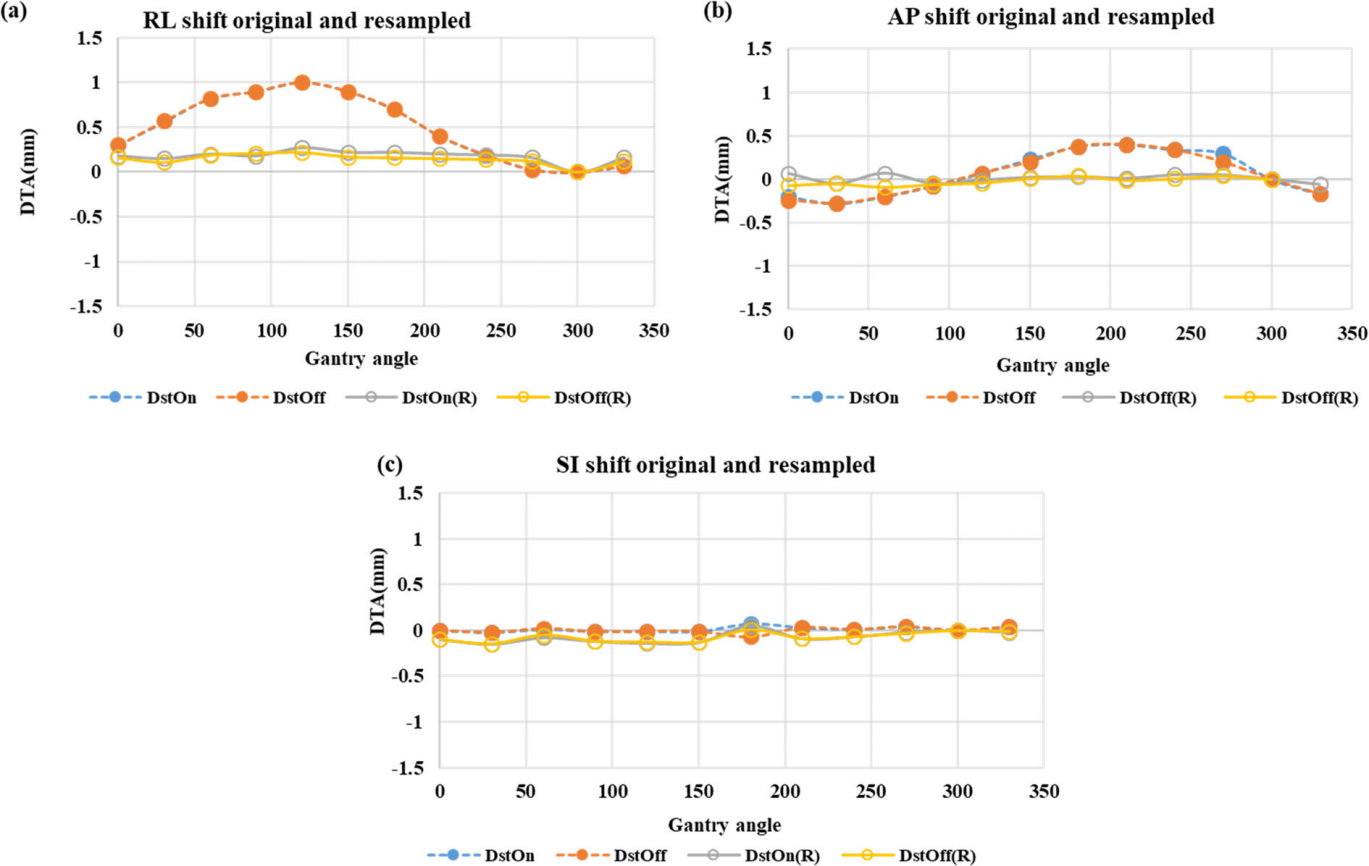
Isocenter shift using MRID^3D^ phantom. The MRI isocenter distance to agreement (DTA) original and resampled for the (a) right–left (RL), (b) anterior–posterior (AP), and (c) superior–inferior (SI) directions across all gantry angles. Each plot includes the value for the distortion correction turned on (DstOn) and off (DstOff) for original images and DstOn(R), DstOff(R) for resampled images.

**FIGURE 5:**
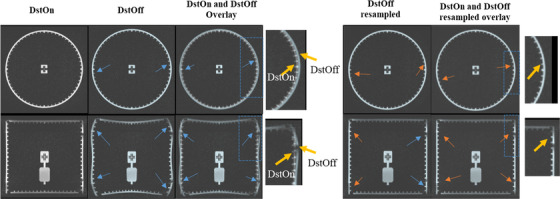
The (a) axial and (b) sagittal view of MRID^3D^ phantom images acquired at a gantry angle of 120° showing the original and resampled images using the extended DVF. The dotted blue square box indicates the overlayed original and resampled images.

As shown in Figure 3, “mean (± SD) distortion” in resampled images of the MRID^3D^ phantom DstOff scan was reduced from 3.2 ± 0.02, 3.0 ± 0.02, and 1.8 ± 0.01 mm to 0.18 ± 0.02, 0.09 ± 0.02, and 0.15 ± 0.01 mm in the AP, SI, and RL directions, respectively. The average “isocenter shift (± SD)” for DstOff scan was reduced from 0.49 ± 0.3, 0.05 ± 0.2, and 0.01 ± 0.03 mm to 0.14 ± 0.05, −0.02 ± 0.04, and −0.07 ± 0.05 mm in the AP, SI, and RL directions, respectively (Figure 4). The original and resampled axial and sagittal images of the MRID^3D^ phantom with DstOn and DstOff conditions acquired at gantry 120° is shown in Figure 5 with the overlay images.

**FIGURE 6:**
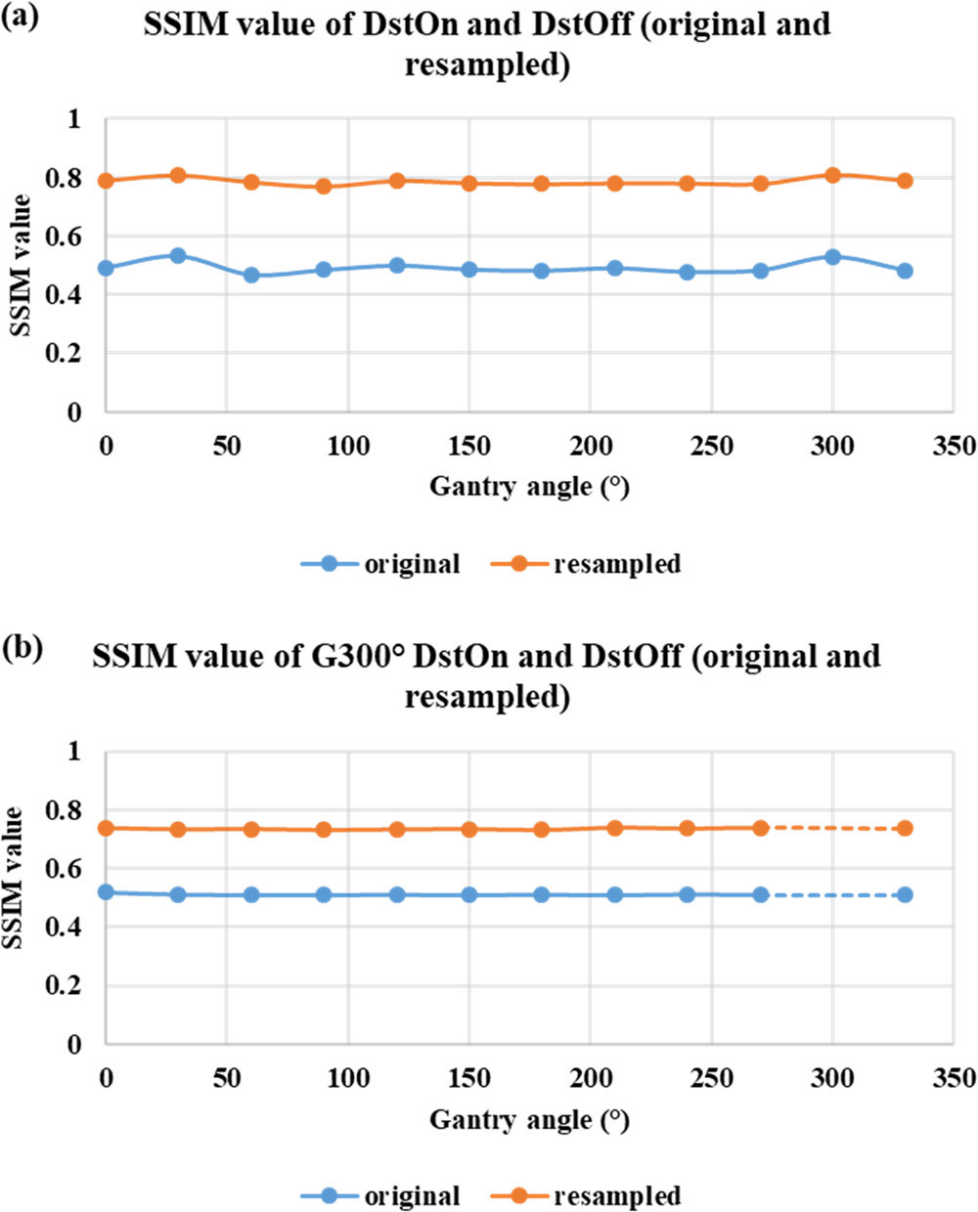
SSIM using MRID^3D^ phantom. (a) SSIM values of DstOff (original and resampled images) compared to DstOn for all measured gantry angles. (b) SSIM values of DstOff (original and resampled images) for all measured gantry angles compared to the baseline G300° DstOn image.

The geometric distortion correction was further evaluated by measuring the SSIM value for original and resampled DstOff images for all gantry angles relative to DstOn images. The average SSIM (± SD) value for original and resampled DstOff images increased from 0.49 ± 0.02 to 0.78 ± 0.01 for all gantry angles relative to DstOn images (Figure 6). Also, the SSIM (± SD) value for original and resampled DstOff images at all gantry angles increased from 0.51 ± 0.05 to 0.73 ± 0.02 relative to DstOn gantry 300° images (Figure 6b).

#### 3.2.2 Independent phantom image correction

**FIGURE 7:**
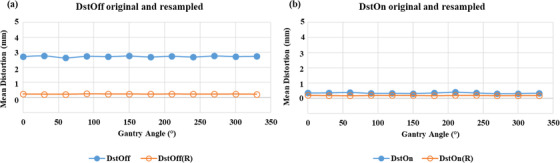
Mean distortion using Fluke phantom. The mean distortion values for original and resampled images across all the gantry angles. The plot shows the values with (a) DstOff original and resampled and (b) DstOn original and resampled.

**FIGURE 8:**
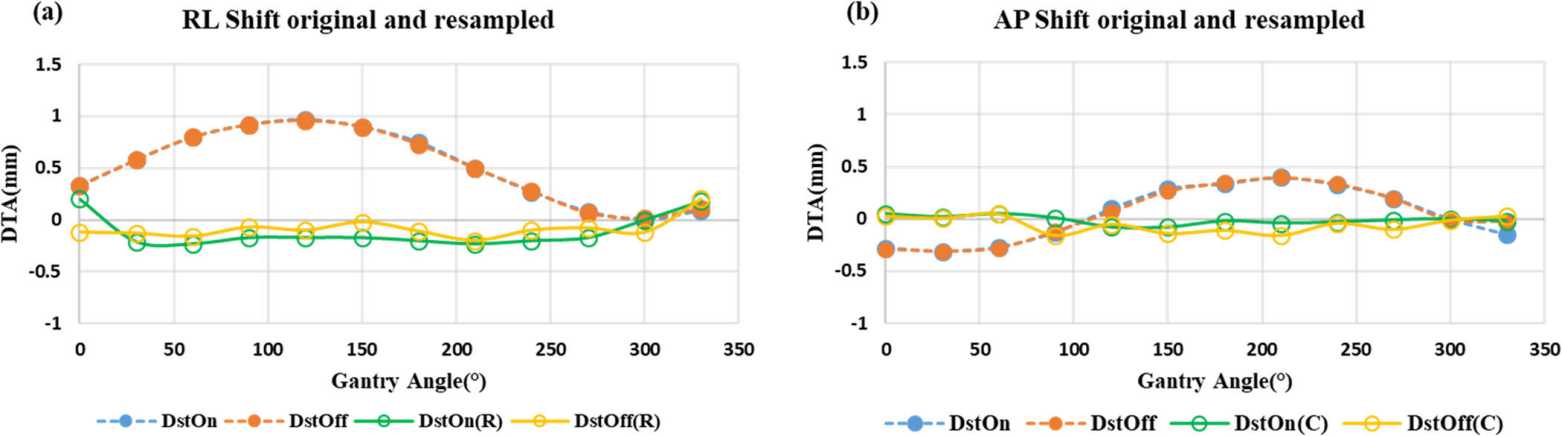
Isocenter shift using Fluke phantom. The MRI isocenter distance to agreement (DTA) original and resampled for the (a) right–left (RL) and (b) anterior–posterior (AP) directions across all gantry angles with the phase encoding in the anterior–posterior (AP) directions. Each plot includes the value for the distortion correction turned on (DstOn) and off (DstOff) for original images and DstOn(R), DstOff(R) for resampled images.

The extended DVFs generated from the MRID^3D^ phantom images for each gantry angle were applied to the Fluke phantom independently to correct its geometric distortion and isocenter shifts. The “mean distortion (± SD)” was corrected from 2.7 ± 0.03 to 0.22 ± 0.01 and 0.33 ± 0.01 mm to 0.18 ± 0.01 mm for DstOff and DstOn scans, respectively (Figure 7). The average “isocenter shift (± SD)” for the DstOff scan was corrected from 0.51 ± 0.03 and 0.05 ± 0.02 mm to −0.08 ± 0. 01 and −0.05 ± 0.07 mm in RL and AP directions, respectively. The average “isocenter shift (± SD)” for the DstOn images was corrected from 0.51 ± 0. 03 and 0.04 ± 0.02 mm to −0.1 ± 0.04 and −0.01 ± 0.01 mm in RL and AP directions, respectively (Figure 8).

**FIGURE 9:**
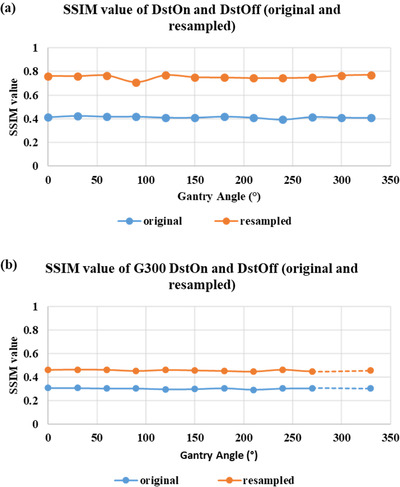
SSIM using Fluke phantom. (a) SSIM values of DstOff (original and resampled images) compared to DstOn for all measured gantry angles. (b) SSIM values of DstOff (original and resampled images) for all measured gantry angles compared to the baseline G300° DstOn image.

As shown in Figure 9a. the average SSIM (± SD) values for the DstOff resampled images increased from 0.41 ± 0.02 to 0.75 ± 0.01 relative to the corresponding DstOn images for all the gantry angles. Similarly, the SSIM (± SD) value for all the gantry angles increased from 0.30 ± 0.03 to 0.45 ± 0.08 for DstOff original and resampled images relative to the baseline DstOn image at gantry 300° (Figure 9b).

#### 3.2.3 Human image correction

**FIGURE 10:**
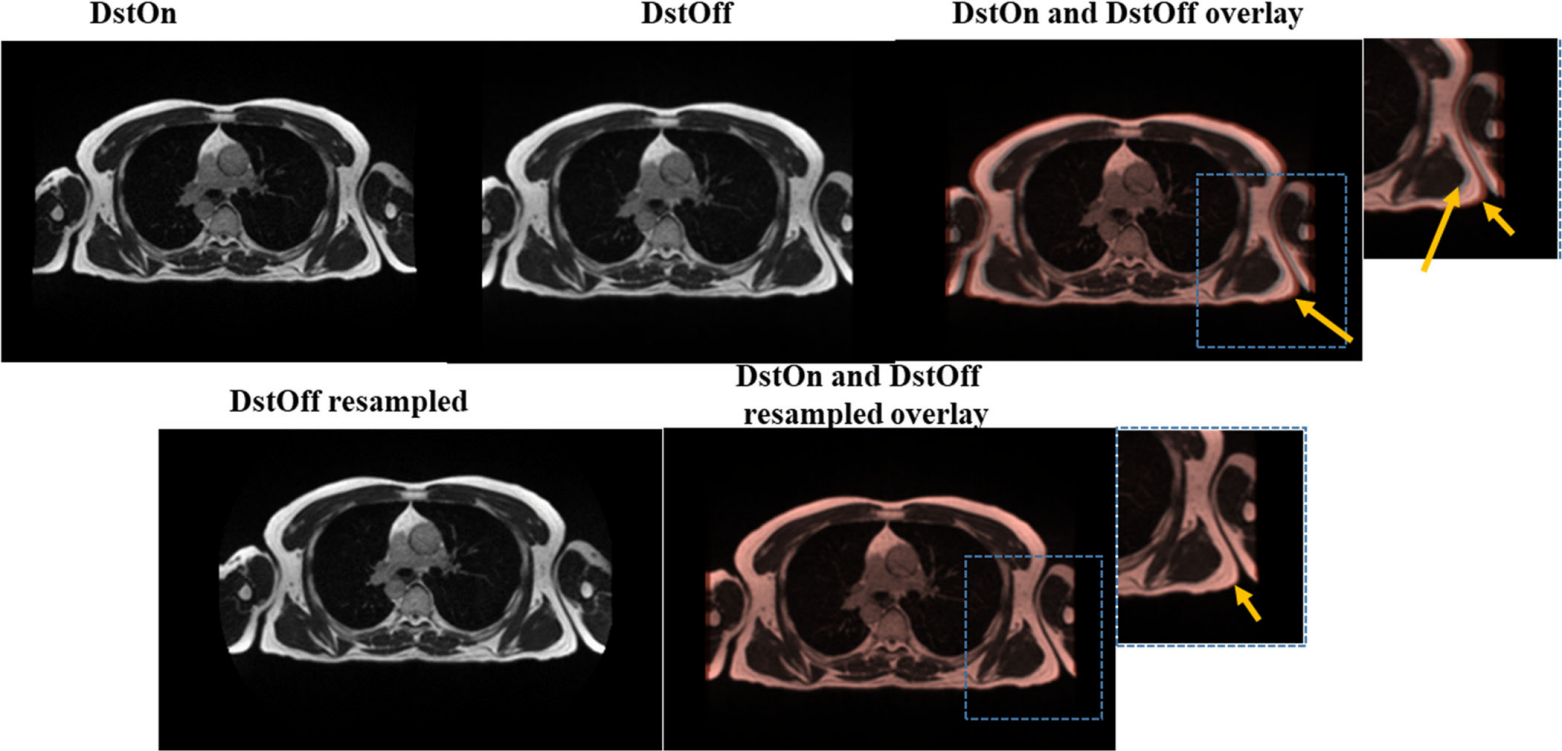
The axial view of the human chest MR image acquired at gantry 300° showing the original image and resampled image using the extended DVF generated from the MRID^3D^ phantom. The images are shown with the onboard Siemens distortion correction function turned on (DstOn) and turned off (DstOff). The dotted blue square box indicates the overlayed original and resampled images. In the overlay images, the primary image (DstOn) upper color is shown in white, and the secondary image (DstOff) upper color is shown in light brown.

The extended DVF generated from the MRID^3D^ phantom images for gantry 300° was applied to the human image to correct its geometric distortion. The original and resampled DstOff and DstOn axial images are displayed in Figure 10. The SSIM of original and resampled DstOff image increased from 0.86 ± 0.25 to 0.98 ± 0.08 relative to DstOn image at gantry 300°.

## 4 DISCUSSIONS

In this study, geometric distortion and the imaging isocenter shift emerging at different gantry positions were characterized using the MRID^3D^ phantom and the associated geometric distortion analysis software on an institutional 0.35 T MR‐Linac system. The MRID^3D^ phantom used in this study is lightweight and cylindrical with high structural rigidity and covers a large imaging FOV in one acquisition without the need for repositioning. Image acquisition over a large FOV is especially important for the MR‐Linac system where geometric accuracy is needed at the image periphery for accurate localization and dose calculation during online adaptive radiotherapy. The systematic investigation for the correction of the geometric distortion and imaging isocenter shift was done by generating DVFs with an extended FOV. The extended DVFs produced were applied to the phantoms images to be corrected back to their original geometry over multiple gantry angles.

The characterization of geometric distortion in MR images using the MRID^3D^ phantom was investigated previously by Lewis et al.^24^ In the study, DVFs were produced to correct the system‐dependent distortion present in the acquired MR images. However, DVFs generated applied only within the phantom FOV (i.e., 343.5 mm diameter and 294 mm length) which is smaller than the physical dimension of the phantom (i.e., 394 mm in diameter and 391 mm in length). Due to the limited FOV, the DVFs produced were not able to cover the peripheral region of the imaging FOV of the phantom, and as a result could not be used to correct the images from which they were produced. Hence, for the systematic verification, the DVFs were applied to the secondary small FOV grid phantom. In this study, using the same spherical harmonic principle used to calculate the DVF within the phantom boundary,^25^ the DVF coverage was extended by 108 mm × 108 mm in diameter and length to a total of 451.5 mm in diameter, and 402 mm in length, which is nearly equivalent to the institutional 0.35 T MRgRT system FOV (450 mm in diameter and 450 mm in length). Thus, the extended DVFs could be used to characterize and correct the image distortion beyond the periphery of the phantom. With the application of the extended DVFs the average geometric distortion (± SD) of the DstOff MRID^3D^ phantom images was corrected from 3.1 ± 0.03 mm to 0.2 ± 0.01 mm which is similar to DstOn geometric distortion (± SD) value 0.2 ± 0.02 mm. This further substantiated the proposed method. The distortion of the DstOn MRID^3D^ phantom images was reduced from 0.31 ± 0.03 to 0.20 ± 0.02 mm, demonstrating that the distortion can be reduced even further by starting with the system‐corrected images. The systematic verification of the imaging distortion correction was done by the re‐characterization of the resampled phantom images.

The independent evaluation of the proposed method was done with the application of extended DVFs to independent phantom images and human abdominothoracic images. The geometric distortion of the independent phantom images at various gantry angles was characterized using its own autonomous software. The extended DVFs produced from MRID^3D^ phantom images were applied to images of the Fluke phantom independently. The average geometric distortion for the images of the independent Fluke phantom was corrected from 2.7 ± 0.1 to 0.2 ± 0.1 mm which is similar to the DstOn geometric distortion (± SD) value 0.33 ± 0.03 mm. The extended DVFs applied to DstOn images further corrects DstOn image geometric distortion (± SD) value 0.33 ± 0.03 to 0.18 ± 0.01 mm. Compared to MRID^3D^ phantom, Fluke phantom image distortion was characterized and corrected only in a single plane. This is due to the structural difference of the phantoms. The respective software of both the phantoms analyzed the image distortion based on their structural components. The proposed technique was further verified with the correction of a human MR image which is shown in Figure 10. A minor geometric distortion present on a human MR image which is barely noticeable in DstOff image was still corrected using the extended DVFs.

The onboard geometric distortion correction is able to remove most distortion present in the peripheral regions of the image, however, it does not correct the variation in the imaging isocenter position due to central frequency shift.^13^, ^14^ In this study, using the proposed method, we characterized and corrected the imaging isocenter shift that occurred at various gantry positions. The isocenter shift was higher at 90–125° gantry angle and in the RL (*x* axis) and AP (*y* axis) direction than in the SI (*z* axis). This was due to the geometrical asymmetry of the rotating gantry.^26^ The maximum isocenter shift of 1 mm was corrected to 0.2 mm for both MRID^3D^ phantom DstOff and DstOn images. The imaging isocenter shift for an independent Fluke phantom was corrected from 0.97 and 0.96 mm to −0.1 mm. The average isocenter‐shift value for the resampled images of Fluke phantoms in AP direction has a higher correction error margin, ranging from 0.05 to −0.05 compared to RL direction (0.51 to −0.08 mm) (Table 6). This is due to the uncertainties for the lower measured values, that is, ≤0.03 mm in different systems such as image resolution, DVF, and analysis softwares.^14^

The DstOff resampled images for the MRID^3D^ phantom, the Fluke phantom, and the human were compared with the vendor‐corrected (DstOn) images. DstOff resampled and DstOn images show a similar extent of geometric distortion correction at all gantry angles. Although the geometric distortion was corrected with the system integrated distortion correction function, the imaging isocenter shift at various gantry angles was not remedied in the DstOn scan. Therefore, in this study, we utilized a single algorithm, that is, use of extended DVFs to resample both the geometric distortion and imaging isocenter shift of DstOff and DstOn scans.

This work has a few limitations. First, the process of characterization and correction of image distortion was done offline. This method could be applied to the online adaptive radiotherapy process with the MRgRT vendor's support. Second, the extended DVFs were limited to 3D volumetric images only, where the extent of geometric distortion and imaging isocenter shift was fixed after the change in each gantry position. In the case of 2D cine imaging, the gantry moves during the imaging so the correction parameters would need to be tabulated and applied in the MRgRT system with the MRgRT vendor's support. In the future, we will be directing the investigation on tracking the imaging isocenter shift and correcting the geometric distortion during the 2D cine imaging using this method. Third, the proposed method in this study is applicable only to system‐dependent distortion, not patient‐dependent distortion. Correction of patient‐related distortion requires more complicated techniques as each patient has a unique set of magnetic properties. Fourth, the proposed method has been tested and verified only on a single institutional 0.35 T MRgRT system. Further testing on other 0.35 T MRgRT systems is required before clinical implementation.

This work demonstrated the clinical possibility of the proposed tool in the correction of the system‐dependent geometric distortion and imaging isocenter shift at different gantry angles through phantom‐based MR images. Furthermore, the correction of the independent phantom images and human MR images provided independent verification of the proposed method.

## 5 CONCLUSIONS

The system‐dependent image distortion characterization and correction method for the 0.35 T MRIdian LINAC system was presented in this work. Extended DVFs with a FOV beyond the MRID^3D^ phantom's boundary were introduced to correct the geometric distortion and the imaging isocenter shift of the phantom images. The extended DVFs generated from the cylindrical MRID^3D^ phantom images were successfully applied to an independent phantom image and also to human MR images on the abdominothoracic region to correct their imaging artifacts, including geometric distortion and isocenter shift.

## AUTHOR CONTRIBUTIONS

Shanti Marasini contributed to data collection, analysis, and wrote the manuscript. Benjamin Quinn, Mike Cole, and Rocco Flores contributed to designing the data analysis system, analysis tool, and provided technical assistance. Taeho Kim contributed to the data collection, analysis, and supervision of the project. All authors discussed the result and contributed to the final manuscript.

## CONFLICT OF INTEREST

All authors declare that they have no conflicts of interest.

## Data Availability

The data that support the findings of this study are available from the corresponding author upon reasonable request.
